# The International Nurses' Council

**Published:** 1909-07-24

**Authors:** 


### THE INTERNATIONAL NURSES' COUNCIL.

### MR. SYDNEY HOLLAND ON ITS NON-REPRESENTATIVE CHARACTER.

In the *Daily Telegraph* of July 21 a letter appeared from the Hon. Sydney Holland, Chairman of the London Hospital, from which we reprint the following extracts. These expose in generous terms the hollow nature of the claims of the "International Council of Nurses"—the promoters of the present International Conference of Nurses, to be representative of English nurses and English hospitals, and to speak authoritatively for British Nursing;—

"I have seen the authorised programme of the English ladies advertised to take a leading part or read a paper. Only one lady is a matron of a large training school, *i.e.*, Miss Stewart, the matron of St. Bartholomew's. The others have little or nothing to do with the training of nurses in England. There is a lady, the matron of a private nursing home and institute at Bournemouth, but described in the programme under the magnificent title of president of the Victoria and Bournemouth Nurses' League! Another is a matron of a hospital in Leicester with about seventy nurses, who is described as president of the Leicester Infirmary Nurses' League. There is a matron from Southampton, a hospital with only about forty nurses, who is described as president of the Royal South Hants Nurses' League. This 'Royal' is good. The name of this lady's hospital is 'The Royal South Hants and Southampton Hospital,' but whatever the hospital may be, the Nurses' League is no more Royal than I am. Then there are three ladies from Ireland, two of whom are certainly not matrons of hospitals, but one of whom is no less than 'vice-president of the National Council of Nurses of Great Britain and Ireland'—that sounds well—and in the chair is Mrs. Bedford Fenwick, late matron of St. Bartholomew's, who is 'president of the International Council of Nurses,' and now editor of a nursing paper which, so the programme says, 'is the official organ of the Congress—price 1d. weekly; please order from your newsagent.'"

"I am only concerned to tell these foreign nurses that but very few matrons, possibly only from three or four London hospitals, will take part in the proceedings of the Congress, and that as regards State registration, no less than sixty-seven matrons of London hospitals, 175 matrons of provincial hospitals, and 1,325 nurses have again quite recently signed a protest against it. These ladies from abroad will find that at all future Congresses, as at all past ones, the same ladies only will appear from England, with or without new titles."

